# Incidence proportion and divergent performance of risk factors for carbapenem-resistant Gram-negative bacteria bloodstream infections across haematological cohorts

**DOI:** 10.1093/jacamr/dlag140

**Published:** 2026-07-22

**Authors:** Guillermo Maestro-de la Calle, María Calbacho, Javier Mateo Flores, Adolfo Jesús Sáez-Marín, Anatolio Alonso-Crespo, Rodrigo Gil-Manso, Celia García-González, Esther Viedma, José Manuel Caro-Teller, Luis Carpeño Fernández, Manuel Lizasoain-Hernández

**Affiliations:** Antimicrobial Stewardship Program in Hematological Patients, 12 de Octubre University Hospital (PROA-HEM HU12O), Gta. Málaga, 11, Usera, Madrid 28041, Spain; Internal Medicine Service, 12 de Octubre University Hospital, Gta. Málaga, 11, Usera, Madrid 28041, Spain; Research Institute Hospital 12 de Octubre (i+12), 12 de Octubre University Hospital, Madrid, Spain; Faculty of Medicine, Complutense University of Madrid, Madrid, Spain; Antimicrobial Stewardship Program in Hematological Patients, 12 de Octubre University Hospital (PROA-HEM HU12O), Gta. Málaga, 11, Usera, Madrid 28041, Spain; Hematology Service, 12 de Octubre University Hospital, Madrid, Spain; Antimicrobial Stewardship Program in Hematological Patients, 12 de Octubre University Hospital (PROA-HEM HU12O), Gta. Málaga, 11, Usera, Madrid 28041, Spain; Internal Medicine Service, 12 de Octubre University Hospital, Gta. Málaga, 11, Usera, Madrid 28041, Spain; Research Institute Hospital 12 de Octubre (i+12), 12 de Octubre University Hospital, Madrid, Spain; Faculty of Medicine, Complutense University of Madrid, Madrid, Spain; Antimicrobial Stewardship Program in Hematological Patients, 12 de Octubre University Hospital (PROA-HEM HU12O), Gta. Málaga, 11, Usera, Madrid 28041, Spain; Research Institute Hospital 12 de Octubre (i+12), 12 de Octubre University Hospital, Madrid, Spain; Faculty of Medicine, Complutense University of Madrid, Madrid, Spain; Hematology Service, 12 de Octubre University Hospital, Madrid, Spain; Internal Medicine Service, 12 de Octubre University Hospital, Gta. Málaga, 11, Usera, Madrid 28041, Spain; Antimicrobial Stewardship Program in Hematological Patients, 12 de Octubre University Hospital (PROA-HEM HU12O), Gta. Málaga, 11, Usera, Madrid 28041, Spain; Hematology Service, 12 de Octubre University Hospital, Madrid, Spain; Internal Medicine Service, 12 de Octubre University Hospital, Gta. Málaga, 11, Usera, Madrid 28041, Spain; Antimicrobial Stewardship Program in Hematological Patients, 12 de Octubre University Hospital (PROA-HEM HU12O), Gta. Málaga, 11, Usera, Madrid 28041, Spain; Research Institute Hospital 12 de Octubre (i+12), 12 de Octubre University Hospital, Madrid, Spain; Microbiology Service, 12 de Octubre University Hospital, Madrid, Spain; Antimicrobial Stewardship Program in Hematological Patients, 12 de Octubre University Hospital (PROA-HEM HU12O), Gta. Málaga, 11, Usera, Madrid 28041, Spain; Research Institute Hospital 12 de Octubre (i+12), 12 de Octubre University Hospital, Madrid, Spain; Pharmacy Service, 12 de Octubre University Hospital, Madrid, Spain; Faculty of Medicine, Complutense University of Madrid, Madrid, Spain; Antimicrobial Stewardship Program in Hematological Patients, 12 de Octubre University Hospital (PROA-HEM HU12O), Gta. Málaga, 11, Usera, Madrid 28041, Spain; Internal Medicine Service, 12 de Octubre University Hospital, Gta. Málaga, 11, Usera, Madrid 28041, Spain; Research Institute Hospital 12 de Octubre (i+12), 12 de Octubre University Hospital, Madrid, Spain; Faculty of Medicine, Complutense University of Madrid, Madrid, Spain; Infectious Diseases Unit, 12 de Octubre University Hospital, Madrid, Spain

## Abstract

**Background:**

There is a lack of standard data on the frequency of carbapenem-resistant Gram-negative bloodstream infections (CR-GNBSI) in haematology wards compared to other hospital units, as well as the factors that increase the risk of acquiring one.

**Methods:**

Retrospective observational study of GNBSI in haematological patients conducted from January 2019 to December 2022. The primary outcome was the incidence proportion (IP) of CR-GNBSI in the haematology ward and its incidence proportion ratio (IPR) compared with the rest of the hospital wards. Secondary outcomes included the identification of risk factors associated with CR-GNBSI in two cohorts: haematological patients with GNBSI and a case–control cohort of febrile neutropenia (FN).

**Results:**

The IP of CR-GNBSI in the haematology ward was 6.12 per 1000 admissions, while its IPR in relation to the rest of the hospital wards was 6.83 (95% CI 3.80–11.46; *P* < 0.001). A composite variable incorporating breakthrough FN, ≥20 days from hospital admission, ≥45 days of hospitalization or ≥7 days of carbapenem therapy, was a strong predictor of CR-GNBSI in the GNBSI cohort (OR 22.9; 95% CI 5.0–105) and in the FN cohort (OR 7.43; 95% CI 1.5–38.8). Its positive predictive value decreased from 26.5% (CI95% 20.5–33.3) in the GNBSI cohort to 2.75% (95% CI 1.92–3.93) in the FN cohort.

**Conclusions:**

The haematology ward has a significantly higher IP of CR-GNBSI compared to the other hospital wards. The performance of risk factors for CR-GNBSI varies significantly depending on the cohort under study.

## Introduction

In recent years, there has been an increasing call for caution in patients with haematological conditions, due to the higher incidence of Gram-negative bloodstream infections (GNBSI) and carbapenem-resistant Gram-negative bloodstream infections (CR-GNBSI).^1^ These infections represent a significant clinical challenge for haematological patients due to the heightened risk of administering inappropriate empirical antibiotic treatment and the ensuing elevated mortality rate.^2–4^ Although several articles consistently report a high incidence of CR-GNBSI in haematology wards, most have focused on absolute numbers rather than standardized incidence measures, limiting meaningful comparisons across hospital settings.^5^

This increased incidence of resistant GNBSI has led to the recommendation of considering novel β-lactams or β-lactamase inhibitors for high-risk febrile neutropenia (FN).^6^ However, the use of broad-spectrum anti-Gram-negative regimens may contribute to the emergence of carbapenem resistance.^7,8^ Furthermore, thereis an increasing incidence of metallo-β-lactamase-producing Enterobacterales, which further limits therapeutic options.^9^ Together, these factors underscore the need for more precise identification of patients truly at risk for CR-GNBSI.

Risk of CR-GNBSI stratification has largely relied on clinical factors derived from heterogeneous patient populations, predominantly outside the haematological setting. Even in this general population, there is an absence of a validated and widely applicable prediction score.^10^ Moreover, extrapolating risk factors across institutions is questionable given the substantial variability in local epidemiology and antimicrobial stewardship practices.^1,11^ Importantly, little is known about how the performance of such risk factors may vary depending on the target population, particularly between FN and GNBSI.

The present study aimed to address two specific knowledge gaps. Firstly, we quantified the incidence proportion (IP) of CR-GNBSI over a 4-year period in the haematology ward and compared it with the IP observed in the rest of the hospital using the incidence proportion ratio (IPR). Secondly, we sought to identify local risk factors for CR-GNBSI in the haematology ward and to evaluate their diagnostic performance in two related but distinct cohorts: haematological patients with GNBSI and those presenting with FN.

## Materials and methods

Retrospective study conducted at a tertiary university hospital, which provides healthcare to a population of 450 000 people, from January 2019 to December 2022. The haematology department runs a dedicated 30-bed inpatient ward, which admitted 2412 patients during the study period. It provides care for patients with haematological malignancies, including haematopoietic stem cell transplantation and cellular therapies, such as CAR-T therapy. The study included haematological patients with GNBSI caused by *Escherichia coli*, *Klebsiella pneumoniae, Enterobacter cloacae*, and *Pseudomonas aeruginosa, occurring either* during hospitalization or in the ambulatory setting.

Two study cohorts were defined. Firstly, patients with confirmed GNBSI. Secondly, patients with FN were analyzed separately. The FN cohort included controls (FN without CR-GNBSI) and cases (CR-GNBSI during FN) in a 3:1 ratio. Controls were randomly selected from all admissions that experienced at least one FN. To minimize temporal selection bias in patients with multiple FN episodes during their hospitalization, a two-step randomization procedure was applied, assigning a random date during hospitalization and then randomly defining the direction of episode selection (towards the admission or towards the discharge). The rationale for performing a separate analysis in the FN cohort was that empirical antibiotic decisions are typically made at the onset of FN, before blood culture results are known.

The primary outcome was the IP of CR-GNBSI in the haematology ward and its IPR in relation to the rest of the hospital wards. Secondary outcomes included the identification of risk factors associated with CR-GNBSI and the evaluation of their diagnostic performance (sensitivity, specificity, positive and negative predictive values) in both the GNBSI cohort and the FN cohort.

An episode of GNBSI was defined as the period from the date of the first isolation of the microorganism until 30 days after the last positive blood culture. CR-GNBSI was defined as resistance to meropenem according to the European Committee on Antimicrobial Susceptibility Testing (EUCAST) clinical breakpoints for each microorganism (v_12.0, 2022).^12^ The microbiological analysis of blood cultures was conducted using the automated BD BACTEC^TM^ blood culture system. Episodes of FN were identified based on the presence of fever (temperature ≥37.8°C), an absolute neutrophil count <500/mm^3^ (or a predicted decrease to <500/mm^3^ within 48 hours),^13^ and blood culture sampling. Breakthrough FN was defined as occurring concomitantly with systemic antibiotic therapy or within 7 days after its completion.^14^ Multidrug-resistant Gram-negative bacteria (MDR-GNB) were defined as isolates non-susceptible to at least one agent in three or more antimicrobial categories.^15^ Anonymized clinical data were collected on the REDCap platform as part of the Antimicrobial Stewardship Program in haematological Patients. The study was granted ethical approval by the local ethics committee (TP24/0292).

IP were calculated as the number of events per 1000 admissions. Comparisons between groups were expressed as IPR and estimated using the exact Poisson method with 95% CI (MedCalc Software Ltd., Ostend, Belgium; available at https://www.medcalc.org/). Comparisons between categorical variables were performed using the chi-square test, and comparisons between continuous variables were conducted using the Mann–Whitney U test.

Risk factors associated with CR-GNBSI were assessed in SPSS v25 using univariate logistic regression analysis, with OR and 95% CI for each factor. Due to the limited sample size, a composite variable was constructed by combining statistically significant (*P* < 0.05) and clinically relevant variables in the risk of CR-GNBSI. This was done in order to preserve relevant clinical information and prediction model stability.^16^ The use of a composite variable is exploratory and does not imply equivalence or equal clinical impact among its individual components. In instances where complete or quasi-complete separation occurred due to the absence of events in one of the comparison groups, Ridge regression was implemented to ensure the stability of coefficient estimation, and penalized estimates were reported. OR and their corresponding 95% CI were derived from the penalized model. For continuous variables, the inspection of sensitivity and specificity values across the receiver operating characteristic (ROC) curve was performed to select cut-off points that maximize sensitivity while minimizing the risk of missing identification of CR-GNBSI. Cut-off points were not selected using an automated optimization criterion. All baseline variables listed in Tables S1 and S2 (available as Supplementary data at *JAC-AMR* Online) were evaluated in univariate logistic regression analyses.

## Results

### Absolute incidence, IP and IPR of GNBSI and CR-GNBSI in the haematology ward and comparison with the rest of the hospital wards

During the 4-year study period, there were 2527 GNBSI, of which 183 (7.2%) occurred in the haematology ward and 2344 (92.8%) in the remaining hospital wards. Of these GNBSI, 104 (56.8%) and 1489 (59.75%) were due to *E. coli* (*P* = 0.649), 31 (16.9%) and 510 (20.5%) to *K. pneumoniae* (*P* = 0.252), 13 (7.1%) and 107 (4.29%) to *E. cloacae* (*P* = 0.077) and 35 (19.1%) and 238 (9.55%) to *P. aeruginosa* (*P* < 0.001), respectively (Table 1). Overall, there were 164 (6.49%) CR-GNBSI, 16 (8.74%) in the haematology ward and 148 (6.31%) in the rest of the hospital wards (*P* = 0.199). There was a higher incidence of CR-*P. aeruginosa* bloodstream infections (BSIs) in the haematology ward (6.01% versus 1.81%; *P* < 0.001).

**Table 1.** Absolute incidence of Gram-negative bloodstream infections between 2019 and 2022 in the haematology ward and comparison with the rest of the hospitalization

	*Overall* *n = 2527*	*HEM* *n = 183 (7.2%)*	*Non-HEM* *n = 2344 (92.8%)*	*P*
*Escherichia coli*	1593 (63.04%)	104 (56.83%)	1489 (59.75%)	0.649
*Klebsiella pneumoniae*	541 (21.41%)	31 (16.94%)	510 (20.47%)	0.252
CR *K. pneumoniae* CR	102 (4.04%)	3 (1.64%)	99 (3.97%)	0.113
*Enterobacter cloacae*	120 (4.75%)	13 (7.10%)	107 (4.29%)	0.077
CR *E. cloacae*	6 (0.24%)	2 (1.09%)	4 (0.16%)	0.011
*Pseudomonas aeruginosa*	273 (10.80%	35 (19.13%)	238 (9.55%)	<0.001
CR *P. aeruginosa*	56 (2.22%)	11 (6.01%)	45 (1.81%)	<0.001
Overall CR-GNB^a^	164 (6.49%)	16 (8.74%)	148 (6.31%)	0.199

HEM, haematology ward; CR,  carbapenem resistant; Non-HEM, overall hospital bloodstream infections excluding the haematology ward.

^a^ *K. pneumoniae, E. cloacae* and *P. aeruginosa* CBP-R. No *E. coli* bloodstream infections were identified in this group.

The total number of admissions was 167 689, of which 2614 (1.6%) were allocated to the haematology ward, and 165 075 (98.4%) to the remaining hospital wards. These data yielded an IP of GNBSI for every 1000 admissions of 39.8 versus 9.02 for *E. coli* (*P* < 0.001), 11.9 versus 3.09 for *K. pneumoniae* (*P* < 0.001), 5.0 versus 0.65 for *E. cloacae* (*P* = 0.004) and 13.4 versus 1.44 for *P. aeruginosa* (*P* < 0.001), respectively (Table 2). The IP of CR-GNBSI was found to be significantly higher in the haematology ward (6.12 versus 0.90, *P* < 0.001) and accounted for an IPR of 6.83 (95% CI 3.80–11.46; *P* < 0.001). This discrepancy was attributable to a higher IP of *P. aeruginosa* BSIs, and to a lesser extent, *E. cloacae* BSIs.

**Table 2.** Incidence proportion of Gram-negative bloodstream infections for every 1000 admissions between 2019 and 2022 in the haematology ward and comparison with the rest of the hospitalization

	*Overall* *n = 2527*	*HEM* *n = 183 (7.2%)*	*Non-HEM* *n = 2344 (92.8%)*	*P*
*Escherichia coli*	9.50	39.8	9.02	<0.001
*Klebsiella pneumoniae*	3.23	11.9	3.09	<0.001
CR *K. pneumoniae*	0.61	1.1	0.60	0.260
*Enterobacter cloacae*	0.72	5.0	0.65	0.004
CR *E. cloacae*	0.04	0.8	0.02	<0.001
*Pseudomonas aeruginosa*	1.63	13.4	1.44	<0.001
CR *P. aeruginosa*	0.33	4.21	0.27	<0.001
Overall CR-GNBSI^a^	0.98	6.12	0.90	<0.001

HEM, haematology ward; CR, carbapenem-resistant; Non-HEM, overall hospital bloodstream infections excluding the haematology ward.

^a^ *K. pneumoniae, E. cloacae* and *P. aeruginosa*. No *E. coli* bloodstream infections were identified in this group.

### Identification of risk factors associated with CR-GNBSI in the cohort of haematological patients with GNBSI

Out of the 16 CR-GNBSI in the haematology ward, 11 (68.8%) were due to *P. aeruginosa*, 2 (12.5%) to *E. cloacae*, and 3 (18.8%) to *K. pneumoniae*. We found no significant differences between CR-GNBSI and carbapenem-susceptible Gram-negative bloodstream infections (CS-GNBSI) in terms of sex, age, haematological disease type or status, Eastern Cooperative Oncology Group (ECOG) Performance Status Scale, Charlson comorbidity index, MDR-GNB colonization, multinational association for supportive care in cancer (MASCC) index or septic shock at BSI onset (Table S1). A higher proportion of CR-GNBSI was due to *P. aeruginosa* compared with CS-GNBSI (68.8% versus 14.4%, *P* < 0.001).

Among the variables significantly associated with CR-GNBSI, we found breakthrough FN as the one more relevant as a single predictor (OR 11.93; 95% CI 3.5–41.1; *P* < 0.001) and the onset of an ambulatory setting as the strongest protector (OR 0.19; 95%CI 0.004–0.86; *P* = 0.031). Other predictor variables significantly associated with CR-GNBSI were related to hospitalization, such as days from hospital admission at BSI onset (OR 1.06; 95% CI 1.01–1.11; *P* = 0.011). While a recent hospital admission was not associated with CR-GNBSI, cumulative hospitalization days in the preceding 3 months were (OR 1.03; 95% CI 1.01–1.05; *P* = 0.014). Further variables were identified as risk factors for CR-GNBSI, including the sum of days of carbapenem therapy in the last 30 days (OR 1.16; 95% CI 1.03–1.30; *P* = 0.013) and previous isolations of multidrug-resistant GNB in samples other than colonization samples (Table 3). For clarity, only variables reaching statistical significance are reported in Table 3. The complete results of the univariate analyses, including non-significant variables, are provided in Table S6.

**Table 3.** Univariate analysis of predictors of CR-GNBSI in the cohort of patients with GNBSI

	*OR (95% CI)*	*P*
BSI onset in an ambulatory setting	0.19 (0.04–0.86)	0.031
Days of admission before BSI onset	1.06 (1.01–1.11)	0.011
Total number of days of prior hospital admission (3 mo)	1.03 (1.01–1.05)	0.014
Breakthrough febrile neutropenia^a^	11.93 (3.5–41.1)	<0.001
Prior antibiotic therapy (within 1 mo)	3.38 (1.19–9.61)	0.022
Total number of days of systemic antibiotic therapy (1 mo)	1.08 (1.03–1.15)	0.005
Prior carbapenem therapy (1 mo)	4.76 (1.65–13.7)	0.004
Total number of days of carbapenem therapy (1 mo)	1.16 (1.03–1.30)	0.013
Previous isolations of MDR-GNB in samples other than colonization samples (6 mo)	4.73 (1.31–17.11)	0.018
Composite variable for the identification of CR-GNBSI	22.9 (5.00–105.5)	<0.001

BSI, bloodstream infection; GNBSI, Gram-negative bloodstream infections; CR-GNBSI, carbapenem-resistant Gram-negative bloodstream infections; MDR-GNB, multi-drug resistant Gram-negative bacteria; FN, febrile neutropenia.

^a^During the episode of febrile neutropenia, the patient presents with fever and clinical fever while still receiving systemic antibiotics at therapeutic doses or has received them in the previous 7 days.

### Identification of a cut-off point according to ROC curves in quantitative variables predictors of CR-GNBSI in the cohort of haematological patients with GNBSI

ROC curves were performed with the following variables: days from hospital admission at BSI onset, the sum of days of hospitalization in the last 3 months and the sum of days of carbapenem therapy in the last 30 days (Figure S1). The AUC values were 0.71 (95% CI 0.58–0.84; *P* = 0.006), 0.66 (95% CI 0.53–0.80; *P* = 0.033) and 0.67 (95% CI 0.51–0.82; *P* = 0.026), respectively.

A cut-off point was selected according to ROC curves, with a focus on sensitivity over specificity, to provide a dichotomic composite variable. The cut-off points are as follows: ≥20 days from hospital admission at BSI onset, ≥45 days of hospitalization in the last 3 months and a ≥7 days of carbapenem therapy in the last 30 days. OR for these dichotomic variables were 5.4 (95% CI 1.6–17.9; *P* = 0.006), 4.4 (95% CI 1.4–13.4; *P* = 0.009) and 4.3 (95% CI 1.2–15.4; *P* = 0.025), respectively.

### Composite variable for the identification of CR-GNBSI in the cohort of haematological patients with GNBSI

We created a composite variable incorporating four variables that were identified as having a higher OR for predicting CR-GNBSI (Table 3). This variable was defined by the presence of any of the following criteria: breakthrough FN, ≥20 days from hospital admission at BSI onset, ≥45 days of hospitalization in the last 3 months and ≥7 days of carbapenem therapy in the last 30 days. The identification of this composite variable yielded a sensitivity of 87.5% (95% CI 61.7–98.5) and a specificity of 76.8% (95% CI 69.5–82.8). The positive predictive value (PPV) was 26.5% (95% CI 20.5–33.3), and the negative predictive value (NPV) was 98.5% (95% CI 94.6–99.6). These values were estimated for a prevalence of CR-GNBSI of 8.74% (16/183) in haematological patients with Gram-negative BSI. The presence of ≥2 or ≥3 of these four variables offered a much higher level of specificity (95% and 97%, respectively). However, this was at the expense of much lower sensitivity (25% and 13%, respectively).

### Identification of risk factors associated with CR-GNBSI in the cohort of haematological patients with FN

Of the 16 cases of BSI due to CR-GNBSI, 15 (93.8%) were observed in the setting of FN. During the study period, 2406 patients were admitted to the haematology ward, and 1001 episodes of FN were identified. These episodes were randomized, and 45 neutropenic fever episodes with no CR-GNBSI were selected as controls.

The majority of CR-GNBSI in the FN cohort were attributable to *P. aeruginosa* (*n* = 10; 66.7%). Compared with controls, FN due to CR-GNBSI was associated with a significantly worse ECOG Performance Status Scale (score ≥3; 26.7% versus 2.2%; *P* = 0.012), and septic shock at FN onset was more prevalent (20.0% versus 0%; *P* = 0.013). No other significant differences were identified between patients with CR-GNBSI and controls, although the relatively small sample size of the FN cohort may have limited the statistical power to detect differences between groups (Table S2).

**Figure 1.**
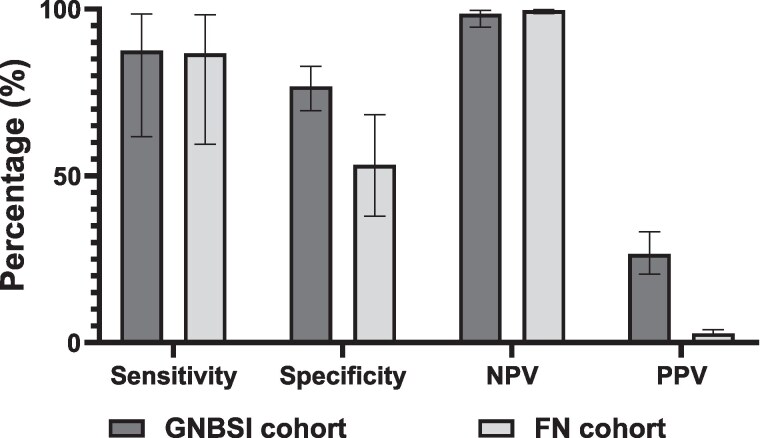
Diagnostic performance of the composite variable for the identification of CR-GNBSI in both cohorts. CR-GNBSI = carbapenem-resistant Gram-negative bloodstream infections; FN = febrile neutropenia; NPV = negative predictive value; PPV = positive predictive value.

In the univariate analysis, the only predictor of CR-GNBSI that was identified was an ECOG Performance Status Scale of ≥3 points (OR 16.0; 95% CI 1.62–157.8; *P* = 0.018). The composite variable for the identification of CR-GNBSI in the GNBSI cohort was also a predictor of CR-GNBSI in the FN cohort (OR 7.43; 95% CI 1.50–38.8; *P* = 0.014) (Table 4). The complete results of the univariate analyses, including non-significant variables, are provided in Table S7. When compared with the GNBSI cohort, the identification of this composite variable in the FN cohort yielded a similar sensitivity (86.7%; 95% CI 59.5–98.3) with a lower specificity (53.3%; 95% CI 37.9–68.34). With a disease prevalence of 1.50% (15/1001), the PPV was 2.75% (95% CI 1.92–3.93), and the NPV was 99.6% (95% CI 98.6–99.9) (Figure 1).

**Table 4.** Univariate analysis of predictors of CR-GNBSI in the cohort of patients with febrile neutropenia

	*OR (95 CI%)*	*P*
ECOG Performance Status Scale upon admission ≥3	16.0 (1.62–157.8)	0.018
Composite variable for the identification of CR-GNBSI^a^	7.43 (1.50–38.8)	0.014

CR-GNBSI, carbapenem-resistant Gram-negative bloodstream infections; ECOG, Eastern Cooperative Oncology Group.

^a^The composite variable was identified when ≥1 of the following was present: breakthrough febrile neutropenia, ≥20 days from hospital admission at bloodstream infection onset, ≥45 days of hospitalization in the last 3 months and ≥7 days of carbapenem therapy in the last 30 days.

## Discussion

Our findings demonstrate that the IPR of CR-GNBSI in the haematology ward in relation to the rest of the hospital is 6.83 (3.80–11.46; *P* < 0.001). This increase was primarily driven by *P. aeruginosa* BSIs. A composite variable was found to be the strongest predictor of CR-GNBSI in the GNBSI cohort (OR 22.9; 95% CI 5.0–105) and in the FN cohort (OR 7.43; 95% CI 1.5–38.8). However, its diagnostic performance decreased significantly in the FN cohort, resulting in a PPV of 2.75% (95% CI 1.92–3.93).

To establish appropriate hospital and haematology ward antibiotic policies, it is essential to understand the absolute and relative incidences of CR-GNBSI. Trecarichi *et al*. reported a total of 74 CR-GNBSI in 15 haematology wards over a period of 3 years in their multicentre study.^17^ Park *et al*. reported 21 CR-GNBSI at their institution over a 19-year period.^2^ These data account for 1.6 and 1.12 CR-GNBSI per haematology ward each year, respectively, and contrast with the incidence of 4.0 CR-GNBSI per year observed at our institution (Table 1). However, this absolute number can be misleading due to variability in admission volumes. The IP, when evaluated in conjunction with the number of admissions, normalizes values for benchmarking and can be used to confirm if CR-GNBSI are more frequent in haematological patients and the real disproportion between them and other hospital wards. Our findings demonstrated that the IPR of CR-GNBSI was significantly higher, at 6-fold, in haematological patients compared with patients on other hospital wards (Table 2). However, large series studies indicate that haematological patients account for less than 5% of the CR-GNBSI, a fact that can divert attention to other populations.^18,19^ Despite the consistent absolute low number of CR-GNBSI cases (ranging from one to four episodes per institution per year), the relative incidence and risk compared with other in-hospital populations is significantly higher, making haematological patients a high priority for infection control and antimicrobial stewardship interventions.

Several risk factors have been associated with carbapenem-resistant Gram-negative bacteria (CR-GNB) infections in haematological patients. Previous colonization or infection with CR-GNB is widely recognized as the most significant risk factor. In our study, only one previous carbapenemase-producing Enterobacteral colonization was identified, and it occurred in a control patient of the FN cohort (Table S3). In contrast, previous isolation of carbapenemase-producing Gram-negative bacteria (CP-GNB) in samples other than those from colonization screening infections was only due to *Pseudomonas aeruginosa* and occurred in only two patients from the CR-GNBSI group in both cohort analyses (Table S4). As previous isolation of CP-GNB would already indicate a need for empirical treatment with novel anti-Gram-negative beta-lactams, these variables were not included in the risk factor analysis for CR-GNBSI. In addition, 12.5%–20% of CR-GNB infections in haematological patients occur in the absence of prior CR-GNB colonization.^20^ Therefore, other risk factors must be considered.

Firstly, previous carbapenem use has been consistently linked to carbapenem-resistant infection or colonization in haematological patients.^4,7,8^ However, none of these studies provided a time breakpoint for use in clinical practice. Conversely, some studies have not found an association between carbapenem use in the previous months and CR-GNBSI.^21^ Nevertheless, it is important to estimate the risk of CR-GNBSI by considering the previous burden of days with carbapenem. Pérez-Galera *et al*. demonstrated in a cohort of hospitalized patients that prior broad-spectrum antibiotics are associated with carbapenem-resistant Enterobacteriaceae as a categorical and time-dependent variable and established ≥6 days as a cut-off for risk association.^18^ This breakpoint is close to the ≥7 days threshold set in our study for the composite variable.

Secondly, our study identified breakthrough FN as the single variable most strongly associated with CR-GNBSI in haematological patients with GNBSI. This could be related to the de-escalation approach at our institution, which is based on meropenem. However, it did not reach significance in the FN cohort (Table S7), which may be due to the dilution of effect when non-bacteremic cases were included, as well as the relatively small sample size limiting statistical power. Previous works have documented that breakthrough FN under carbapenem treatment is an independent predictor of CR-GNBSI with an OR of 9.08 (4.57–18.02), which is similar to the one observed in our GNBSI cohort (Table 3).^22^ Furthermore, Nam *et al*. reported in their FN cohort that CR-GNBSI was only present in patients with breakthrough FN.^14^ All these findings call into question the significance of empirical antibiotic regimens in FN and the recent trends advocating for early de-escalation strategies.^23^

Thirdly, the number of days of admission was also found to be associated with CR-GNBSI (Table 3). The breakpoints selected for the composite variable were set at ≥20 days of admission before BSI, or ≥45 days of admission during the last 3 months. While these lengths of admission may appear excessive, they are consistent with those described by Gallardo-Pizarro *et al*.^20^ They reported a delay of 7 days (IQR 3–19) from admission to CR-GNB colonization and a further 14.5 or 58.5 days to BSI in patients who developed carbapenem-resistant *K. pneumoniae* or *P. aeruginosa* with difficult-to-treat resistance, respectively. As shown in Table 3, each additional day of admission is associated with an increased risk of CR-GNBSI, with OR similar to those previously documented (1.01; 95% CI 1.00–1.02).^22^

Finally, we demonstrate the differential performance of risk factors for CR-GNBSI in two related yet distinct cohorts: haematological patients with GNBSI or with FN. The much lower prevalence in the latter cohort means that our composite variable, which may seem promising in the GNBSI cohort, would lead to an inappropriate use of novel antibiotics with activity against CR-GNB in 97.25% of cases in the FN cohort, with the consequent risk of antimicrobial resistance development. Consequently, we recommend that future studies consider the risk factors in the whole target population (and not just BSIs) if they are to be used in clinical practice. An alternative option would be a two-step approach using risk factors for CR-GNBSI developed in patients with GNBSI after an initial risk assessment of GNBSI using a validated score.^24^

Our study has several limitations. Due to the limited number of outcome events, multivariate logistic regression could not be reliably performed. We developed a composite variable with a meaningful grouping method, which can be considered as an alternative approach to overcome this limitation without incurring excessive loss of degrees of freedom.^16,25^ The composite variable was found to be strongly and significantly associated with CR-GNBSI in both the GNBSI cohort and the FN cohort (Tables 3 and 4). Nevertheless, it should be noted that this composite variable approach does not reflect the individual clinical impact of each variable. It should therefore be taken as exploratory, hypothesis-generating for future studies and is not intended for direct clinical application. Another limitation of our study is that CR-GNBSI were primarily driven by *P. aeruginosa*. This microorganism is characterized by distinct resistance mechanisms and therapeutic challenges. While analyzing *P. aeruginosa* as a separate outcome would be clinically relevant, the limited number of carbapenem-resistant events precluded a reliable stratified analysis. However, carbapenem-resistant Enterobacteriaceae (CRE) are becoming the most prevalent CR-GNB in many haematology wards.^1^ In line with the clinical importance of these pathogens, the World Health Organization has recently updated its Bacterial Priority Pathogens List, prioritizing CRE over *P. aeruginosa* in its 2024 revision.^26^ Detailed antimicrobial susceptibility profiles and carbapenemase characterization were available for the isolated pathogens (Table S5). Therefore, although CR-GNBSI was analyzed as a composite outcome, our findings should be interpreted within this epidemiological context and require to be externally validated in haematology wards where CRE could be the dominant CR-GNBSI.

This study has important clinical implications. We report for the first time the IP of CR-GNBSI in a haematology ward and demonstrate that it is markedly higher than in the rest of the hospital. This finding reflects the burden of CR-GNBSI in this population and highlights the importance of antimicrobial stewardship and infection control in haematology wards.^27^

This study has important clinical implications. We report for the first time the IP of CR-GNBSI in a haematology ward and demonstrate that it is markedly higher than in the rest of the hospital. This finding may explain why antimicrobial stewardship interventions and infection control measures have a higher impact in reducing the incidence of infection and colonization with antibiotic-resistant bacteria in haematology–oncology wards.^27^ Ultimately, the comparison of risk factors for CR-GNBSI indicated how the PPV can dramatically drop from bacteremic patients to FN patients. This should call into caution when applying them in clinical practice to prevent the misuse of novel antibiotics with activity against CP-GNB. The composite variable proposed in our manuscript represents a methodological necessity in the context of limited outcome events, and it should be noted that it is not intended for direct use in clinical practice.

## Supplementary Material

dlag140_Supplementary_Data

## Supplementary data

Figure S1 and Tables S1 to S7 are available as Supplementary data at *JAC-AMR* Online.
