# Supplementary material for: Incidence proportion and divergent performance of risk factors for carbapenem-resistant Gram-negative bacteria bloodstream infections across haematological cohorts
Source: JAC Antimicrob Resist. 2026 Jul 22;8(4):dlag140. doi: 10.1093/jacamr/dlag140 (PMC13390927; doi:10.1093/jacamr/dlag140)
Supplement: dlag140_Supplementary_Data [file dlag140_supplementary_data.docx]

## Supplementary material

| **Table S1**. Baseline characteristics of the cohort of patients with Gram-negative bloodstream infections | | | | |
| --- | --- | --- | --- | --- |
|  | **Overall**  **N = 183** | **CS-GNBSI**  **n = 167** | **CR-GNBSI**  **n = 16** | **p** |
| Microorganism ^a^ |  |  |  |  |
| *Escherichia coli* | 104 (56.8) | 104 (64.2) | 0 | < 0.001 |
| *Klebsiella pneumoniae* | 31 (16.9) | 28 (16.8) | 3 (18.8) | 0.737 |
| *Enterobacter cloacae* | 13 (7.1) | 11 (6.6) | 2 (12.5) | 0.317 |
| *Pseudomonas aeruginosa* | 35 (19.1) | 24 (14.4) | 11 (68.8) | < 0.001 |
| Sex (male) ^a^ | 94 (51.4) | 83 (49.7) | 11 (68.8) | 0.145 |
| Age (years) ^b^ | 59 (48-67) | 59 (48-67) | 55 (47-62) | 0.246 |
| Hematological disease ^a^ |  |  |  |  |
| Acute leukemia/myelodysplastic syndrome | 100 (54.6) | 90 (53.9) | 10 (62.5) | 0.509 |
| Lymphoma | 40 (21.9) | 38 (22.8) | 2 (12.5) | 0.529 |
| Multiple myeloma/Waldenström disease | 31 (16.9) | 29 (17.4) | 2 (12.5) | 1.000 |
| Other | 12 (6.6) | 10 (6.0) | 2 (12.5) | 0.282 |
| Hematological disease status ^a^ |  |  |  |  |
| First line of treatment | 34 (18.6) | 30 (18.0) | 4 (25.0) | 0.503 |
| Refractory disease | 27 (14.8) | 23 (13.8) | 4 (25.0) | 0.262 |
| 1^st^ complete remission | 63 (34.4) | 59 (35.3) | 4 (25.0) | 0.406 |
| ≥2^nd^ complete remission | 26 (14.2) | 24 (14.4) | 2 (12.5) | 1.000 |
| Others | 33 (18.0) | 31 (18.6) | 2 (12.5) | 0.771 |
| HSCT ^a^ | 69 (37.7) | 66 (39.5) | 3 (18.8) | 0.101 |
| ECOG Performance Status Scale upon admission >=3 ^a^ | 29 (15.8) | 25 (15.0) | 4 (25.0) | 0.289 |
| Charlson comorbidity index ^b^ | 0 (0 - 1) | 0 (0 - 1) | 1 (0 - 1.8) | 0.136 |
| MASCC index < 21 ^a^ | 87 (47.5) | 79 (47.3) | 8 (50.0) | 0.854 |
| Septic shock at BSI onset ^a^ | 28 (15.3) | 25 (15.0) | 3 (18.8) | 0.717 |
| BSI onset in an ambulatory setting ^a^ | 74 (40.4) | 72 (43.1) | 2 (12.5) | 0.017 |
| Days of admission before BSI onset ^b^ | 11 (0 - 16) | 10 (0 - 16) | 14.5 (12 - 20.8) | 0.004 |
| Previous neutropenia (previous 90 days) ^a^ | 120 (65.6) | 107 (64.1) | 13 (81.3) | 0.167 |
| Previous febrile neutropenia ^a^ | 69 (37.7) | 60 (35.9) | 9 (56.3) | 0.109 |
| Days from previous febrile neutropenia ^b^ | 90 (49-90) | 90 (51-90) | 65 (24-90) | 0.075 |
| Sum of days of neutropenia (previous 3 mo) ^b^ | 7.5 (0 - 24.0) | 5.5 (0 - 23.0) | 20 (7.0 - 35.3) | 0.045 |
| Neutropenia at onset of BSI ^a^ | 142 (77.6) | 127 (76.0) | 15 (93.8) | 0.127 |
| Neutrophils at the onset of bacteriemia <100/mm^3^ ^a^ | 123 (67.2) | 109 (65.3) | 14 (87.5) | 0.070 |
| Days of neutropenia prior to onset of BSI ^b^ | 5 (0 - 9) | 3 (0 - 9) | 9 (5.5 - 11.5) | 0.010 |
| Breakthrough febrile neutropenia ^a,c^ | 14 (7.7) | 8 (4.8) | 6 (37.5) | < 0.001 |
| Prior antibiotic therapy (within 1 mo) ^a^ | 55 (30.1) | 46 (27.5) | 9 (56.3) | 0.023 |
| Total number of days of systemic antibiotic therapy (1 mo) ^b^ | 0 (0 - 5) | 0 (0 - 4.0) | 6 (0 - 17.5) | 0.006 |
| Prior carbapenem therapy (1 mo) ^a^ | 37 (20.2) | 29 (17.4) | 8 (50.0) | 0.005 |
| Total number of days of carbapenem therapy (1 mo) ^b^ | 0 (0 - 0) | 0 (0 - 0) | 2 (0 - 6.8) | 0.001 |
| Prior hospital admission (3 mo) ^a^ | 98 (53.6) | 89 (53.3) | 9 (56.3) | 0.821 |
| Total number of days of prior hospital admission (3 mo) | 10 (0 - 30) | 10 (0 - 30) | 20 (9.3 - 52.5) | 0.027 |
| Colonization with multidrug-resistant GNB (6 mo) ^a,d^ | 12 (6.6) | 11 (8.4) | 1 (6.3) | 1.0 |
| Previous isolations of MDR-GNB in samples other than colonization samples (6 mo) ^a,d^ | 15 (8.2) | 11 (6.6) | 4 (25.0) | 0.030 |
| BSI = bloodstream infection; CS = carbapenem susceptible; CR = carbapenem resistant; ECOG = Eastern Cooperative Oncology Group; GNB = Gram-negative bacteria; GNBSI = Gram-negative bloodstream infection; MASCC = Multinational association for supportive care in cancer; MDR, multi-drug resistant; HSCT, Hematopoietic stem cell transplantation.  ^a^ Qualitative variable, absolute number (percentage) is shown.  ^b^ Quantitative variable, median (IQR) is shown.  ^c^ During the episode of febrile neutropenia, the patient presents with fever and clinical fever while still receiving systemic antibiotics at therapeutic doses or has received them in the previous 7 days.  ^d^ Details of the MDR-GNB colonization and isolation in other samples are provided in Tables S3 and S4.  Categorical variables were compared using the chi-square test, and continuous variables were compared using the Mann–Whitney U test. | | | | |

| **Table S2**. Baseline characteristics of the cohort of patients with febrile neutropenia | | | | |
| --- | --- | --- | --- | --- |
|  | **Overall**  **N = 60** | **Controls**  **n = 45** | **CR-GNBSI**  **n = 15** | **p** |
| Bloodstream infection ^a^ | 24 (40.0) | 9 (20.0) | 15 (100) | < 0.001 |
| *Escherichia coli* | 2 (3.3) | 2 (4.4) | 0 | 1.00 |
| *Klebsiella pneumoniae* | 4 (6.7) | 1 (2.2) | 3 (20.0) | 0.045 |
| *Enterobacter cloacae* | 2 (3.3) | 0 | 2 (13.3) | 0.059 |
| *Pseudomonas aeruginosa* | 10 (16.7) | 0 | 10 (66.7) | < 0.001 |
| Other Gram-negative bacteria | 1 (1.7) | 1 (2.2) | 0 | 1.00 |
| Gram-positive cocci | 3 (5.0) | 3 (6.7) | 0 | 0.566 |
| Sex (male) ^a^ | 40 (66.7) | 29 (64.4) | 11 (73.3) | 0.527 |
| Age (years) ^b^ | 56.5 (49.3-65) | 57 (49.5-65.5) | 55 (46-64) | 0.468 |
| Hematological disease ^a^ |  |  |  |  |
| Acute leukemia/myelodysplastic syndrome | 35 (58.3) | 25 (55.6) | 10 (66.7) | 0.450 |
| Lymphoma | 19 (31.7) | 17 (37.8) | 2 (13.3) | 0.112 |
| Multiple myeloma/Waldenström disease | 2 (3.3) | 1 (2.2) | 1 (6.7) | 0.441 |
| Other | 4 (6.7) | 2 (4.4) | 2 (13.3) | 0.258 |
| Hematological disease status ^a^ |  |  |  |  |
| First line of treatment | 15 (25.4) | 11 (25.0) | 4 (26.7) | 1.00 |
| Refractory disease | 10 (16.9) | 6 (13.6) | 4 (26.7) | 0.257 |
| 1^st^ complete remission | 25 (42.4) | 21 (47.7) | 4 (26.7) | 0.154 |
| ≥2^nd^ complete remission | 3 (5.1) | 1 (2.3) | 2 (13.3) | 0.156 |
| Others | 6 (10.2) | 5 (11.4) | 1 (6.7) | 1.00 |
| HSCT ^a^ | 4 (22.2) | 1 (33.3) | 3 (20.0) | 1.00 |
| ECOG Performance Status Scale upon admission >=3 ^a^ | 5 (8.3) | 1 (2.2) | 4 (26.7) | 0.012 |
| Charlson comorbidity index ^b^ | 0 (0 - 1) | 0 (0 - 1) | 1 (0 - 1) | 0.387 |
| MASCC index < 21 ^a^ | 31 (51.7) | 21 (46.7) | 10 (66.7) | 0.179 |
| Septic shock at BSI or FN onset ^a^ | 3 (5.0) | 0 | 3 (20.0) | 0.013 |
| FN episode onset in an ambulatory setting ^a^ | 14 (23.3) | 12 (26.7) | 2 (13.3) | 0.483 |
| Days of admission before FN onset ^b^ | 13 (0.75 – 19.0) | 13 (0 - 19) | 14.0 (12 - 20.0) | 0.334 |
| Previous neutropenia (previous 90 days) ^a^ | 53 (88.3) | 40 (88.9) | 13 (86.7) | 1.00 |
| Previous FN episode ^a^ | 33 (55.0) | 24 (53.3) | 9 (60.0) | 0.653 |
| Days from previous FN episode ^b^ | 23 (2.5 - 40.5) | 20 (1-38.5) | 26 (11-49.0) | 0.922 |
| Sum of days of neutropenia (previous 3 mo) ^b^ | 16 (5 – 29) | 16 (5 - 24) | 27 (10 - 37) | 0.239 |
| Neutrophils at the onset of FN episode <100/mm^3^ ^a^ | 59 (98.3) | 44 (97.8) | 15 (100) | 1.00 |
| Days of neutropenia prior to onset of FN episode ^b^ | 16 (5 – 28.5) | 16 (4.5 – 24.0) | 27 (10.0 – 37.0) | 0.212 |
| Breakthrough FN episode ^a,c^ | 19 (31.7) | 13 (28.9) | 6 (40.0) | 0.525 |
| Prior antibiotic therapy (within 1 mo) ^a^ | 30 (50.0) | 22 (48.9) | 8 (53.3) | 0.766 |
| Total number of days of systemic antibiotic therapy (1 mo) ^b^ | 0.5 (0 – 8.5) | 0 (0 – 6.0) | 5 (0 – 20) | 0.307 |
| Prior carbapenem therapy (1 mo) ^a^ | 28 (46.7) | 21 (46.7) | 7 (46.7) | 1.00 |
| Total number of days of carbapenem therapy (1 mo) ^b^ | 0 (0 - 5) | 0 (0 - 5) | 0 (0 - 6) | 0.846 |
| Prior hospital admission (3 mo) ^a^ | 45 (75.0) | 36 (80.0) | 9 (60.0) | 0.169 |
| Total number of days of prior hospital admission (3 mo) | 21 (10 – 40.3) | 23 (11.5 – 35.5) | 20 (9.0 - 55.0) | 0.838 |
| Colonization with multidrug-resistant GNB (6 mo) ^a,d^ | 8 (13.3) | 7 (15.5) | 1 (6.7) | 0.667 |
| Previous isolations of MDR-GNB in samples other than colonization samples (6 mo) ^a,d^ | 8 (13.3) | 5 (11.1) | 3 (20.0) | 0.400 |
| BSI = bloodstream infection; CR = carbapenem resistant; ECOG = Eastern Cooperative Oncology Group; FN = febrile neutropenia; GNB = Gram-negative bacteria; GNBSI = Gram-negative bloodstream infection; MASCC = Multinational association for supportive care in cancer; MDR = multi-drug resistant; HSCT = Hematopoietic stem cell transplantation.  ^a^ Qualitative variable, absolute number (percentage) is shown.  ^b^ Quantitative variable, median (IQR) is shown.  ^c^ During the episode of febrile neutropenia, the patient presents with fever and clinical fever while still receiving systemic antibiotics at therapeutic doses or has received them in the previous 7 days.  ^d^ Details of the MDR-GNB colonization and isolation in other samples are provided in Tables S3 and S4.  Categorical variables were compared using the chi-square test, and continuous variables were compared using the Mann–Whitney U test. | | | | |

| **Table S3**. Colonization with multidrug-resistant Gram-negative bacteria | | | |
| --- | --- | --- | --- |
| **Cohort of patients with Gram-negative bloodstream infection** | | | |
|  | **Total**  **n = 183** | **CS-GNBSI**  **n = 167** | **CR-GNBSI**  **n = 16** |
| *Escherichia coli* ESBL | 9 (4.9) | 8 (4.8) | 1 (6.3) |
| *Klebsiella pneumoniae* ESBL | 1 (0.5) | 1 (0.6) | 0 |
| *Klebsiella variicola* MDR^a^ | 1 (0.5) | 1 (0.6) | 0 |
| *Klebsiella ocytoca* MDR^a^ | 1 (0.5) | 1 (0.6) | 0 |
| Colonization with any multidrug-resistant GNB in the previous 6 months | 12 (6.6) | 11 (8.4) | 1 (6.3) |
| **Cohort of patients with febrile neutropenia** | | | |
|  | **Total**  **N = 60** | **Controls**  **n = 45** | **CR-GNB**  **n = 15** |
| *Escherichia coli* ESBL | 4 (6.7) | 3 (6.7) | 1 (6.7) |
| *Klebsiella pneumoniae* ESBL | 3 (5.0) | 3 (6.7) | 0 |
| *Citrobacter freundii* MDR^a^ | 1 (0.5) | 1 (2.2) | 0 |
| *Enterobacter cloacae* CP | 1 (0.5) | 1 (2.2) | 0 |
| Colonization with any multidrug-resistant GNB in the previous 6 months | 8 (13.3) | 7 (15.5) | 1 (6.7) |
| CP = Carbapenemase-producing; CR = Carbapenem-resistance; CS = Carbapenem susceptible; ESBL = Extended-spectrum beta-lactamases; GNBSI = Gram-negative bloodstream infection; MDR = Multidrug Resistance  ^a^ Phenotypic resistance to ceftazidime and no resistant mechanism specified. | | | |

| **Table S4**. Previous isolations of multidrug-resistant Gram-negative in samples other than colonization samples | | | |
| --- | --- | --- | --- |
| **Cohort of patients with Gram-negative bloodstream infection** | | | |
|  | **Total**  **n = 183** | **CS-GNBSI**  **n = 167** | **CR-GNBSI**  **n = 16** |
| *Escherichia coli* ESBL | 5 (2.7) | 3 (1.8) | 2 (12.5) |
| *Klebsiella pneumoniae* ESBL | 3 (1.6) | 3 (1.8) | 0 |
| *Pseudomonas aeruginosa CP* | 7 (3.8) | 5 (3.0) | 2 (12.5) |
| Previous isolations of MDR-GNB in samples other than colonization samples (6 mo) ^a^ | 15 (8.2) | 11 (6.6) | 4 (25.0) |
| **Cohort of patients with febrile neutropenia** | | | |
|  | **Total**  **N = 60** | **Controls**  **n = 45** | **CR-GNBSI**  **n = 15** |
| *Escherichia coli* ESBL | 4 (6.7) | 3 (6.7) | 1 (6.7) |
| *Klebsiella pneumoniae* ESBL | 1 (1.7) | 1 (2.2) | 0 |
| *Pseudomonas aeruginosa CP* | 2 (3.3) | 0 | 2 (13.4) |
| *Enterobacter cloacae* ESBL | 1 (1.7) | 1 (2.2) | 0 |
| Previous isolations of MDR-GNB in samples other than colonization samples (6 mo) | 8 (13.3) | 5 (11.1) | 3 (20.0) |
| CP = Carbapenemase-producing; CR = Carbapenem-resistance; CS = Carbapenem susceptible; ESBL = Extended-spectrum beta-lactamases; GNBSI = Gram-negative bloodstream infection; MDR, Multidrug Resistance | | | |

| Table S5. Antimicrobial susceptibility profiles and carbapenemase characterization of carbapenem‑resistant Gram‑negative bloodstream isolates | | | | | | | | | | | | | | | | | | |
| --- | --- | --- | --- | --- | --- | --- | --- | --- | --- | --- | --- | --- | --- | --- | --- | --- | --- | --- |
| Microorganism | **FN** | **CRO** | **CEF** | **CAZ** | **PIP/TAZ** | **ETP** | **MEM** | **AZT** | **CIP** | **AMK** | **COL** | **TIG** | **TMX** | **FOS** | **CFDC** | **CAZ/AVI** | **TOL/TAZ** | **CBP** |
| *P. aeruginosa* | Yes | R | R | R | R | R | R | S | R | R | S | R | R | R | NT | S | R | GES |
| *P. aeruginosa* | Yes | R | R | R | R | R | R | R | R | R | S | R | R | R | NT | R | R | GES |
| *P. aeruginosa* | Yes | R | R | R | R | R | R | S | R | R | S | R | R | R | NT | S | R | GES |
| *P. aeruginosa* | Yes | R | R | R | R | R | R | R | R | R | S | R | R | R | NT | S | R | GES |
| *P. aeruginosa* | Yes | R | S | S | S | R | R | S | S | S | S | R | R | R | NT | S | R | GES |
| *P. aeruginosa* | Yes | R | R | R | R | R | R | R | R | R | S | R | R | R | NT | S | R | GES |
| *P. aeruginosa* | Yes | R | R | R | R | R | R | S | R | R | S | R | R | R | NT | S | R | GES |
| *P. aeruginosa* | Yes | R | R | I | I | R | R | R | I | S | S | R | R | R | NT | S | S | Absent |
| *P. aeruginosa* | Yes | R | R | R | R | R | R | R | R | R | R | R | R | R | NT | S | R | GES |
| *P. aeruginosa* | No | R | R | R | R | R | R | I | R | R | S | R | R | R | NT | S | R | GES |
| *P. aeruginosa* | Yes | R | R | I | I | R | R | I | R | R | S | R | R | R | NT | S | S | NT |
| *K. pneumoniae* | Yes | R | R | R | R | R | R | R | R | R | S | S | S | S | NT | S | R | OXA-48 |
| *K. pneumoniae* | Yes | R | R | R | R | R | R | R | R | S | S | S | R | R | NT | S | R | OXA-48 |
| *K. pneumoniae* | Yes | R | R | R | R | R | I | R | R | S | R | R | R | R | NT | S | R | OXA-48 |
| *E. cloacae* | Yes | R | R | R | R | R | R | R | R | S | R | S | R | R | R | R | R | VIM/KPC |
| *E. cloacae* | Yes | R | R | R | R | R | S | R | R | I | S | S | R | NT | NT | R | NT | VIM |
| R = resistant; S = susceptible; I = intermediate susceptibility, according to the corresponding European Committee on Antimicrobial Susceptibility Testing (EUCAST) breakpoints.  CBP = carbapenemase; CRO = ceftriaxone; CEF = cefepime; CAZ = ceftazidime; PIP/TAZ = piperacillin–tazobactam; ETP = ertapenem; FN = febrile neutropenia; MEM = meropenem; AZT = aztreonam; CIP = ciprofloxacin; AMK = amikacin; COL = colistin (polymyxin E); NT = Not tested; TIG = tigecycline; TMX = trimethoprim–sulfamethoxazole; FOS = fosfomycin; CFDC = cefiderocol; CAZ/AVI = ceftazidime–avibactam; TOL/TAZ = ceftolozane–tazobactam | | | | | | | | | | | | | | | | | | |

| **Table S6**. Univariate analysis of baseline variables associated with carbapenem‑resistant Gram‑negative bloodstream infections in the cohort of patients with GNBSI | | |
| --- | --- | --- |
|  | **OR (95% CI)** | **p** |
| Sex (male) | 2.23 (0.74-6.69)7 | 0.154 |
| Age (years) | 0.98 (0.95 – 1.02) | 0.281 |
| Hematological disease |  |  |
| Acute leukemia/myelodysplastic syndrome | 1.43 (0.50-4.10) | 0.511 |
| Lymphoma | 0.49 (0.11-2.23) | 0.352 |
| Multiple myeloma/Waldenström disease | 0.68 (0.15-3.15) | 0.622 |
| Other | 2.24 (0.45-11.3) | 0.327 |
| Hematological disease status |  |  |
| First line of treatment | 1.52 (0.46-5.05) | 0.492 |
| Refractory disease | 2.09 (0.62-7.03) | 0.235 |
| 1^st^ complete remission | 0.61 (0.19-1.98) | 0.410 |
| ≥2^nd^ complete remission | 0.85 (0.18-3.98) | 0.838 |
| Others | 0.63 (0.14-2.90) | 0.550 |
| HSCT | 0.35 (0.10-1.29) | 0.115 |
| ECOG Performance Status Scale upon admission >=3 | 1.89 (0.57-6.34) | 0.301 |
| Charlson comorbidity index | 1.18 (0.89-1.56) | 0.242 |
| MASCC index < 21 | 1.10 (0.40-3.07) | 0.854 |
| Septic shock at BSI onset | 1.30 (0.35-4.90) | 0.697 |
| BSI onset in an ambulatory setting | 0.19 (0.04-0.86) | 0.031 |
| Days of admission before BSI onset | 1.06 (1.01-1.11) | 0.011 |
| Prior hospital admission (within 3 mo) | 1.13 (0.40-3.17) | 0.821 |
| Total number of days of prior hospital admission (3 mo) | 1.03 (1.01-1.05) | 0.014 |
| Previous neutropenia (previous 90 days) | 2.43 (0.67-8.87) | 0.179 |
| Previous febrile neutropenia | 2.29 (0.81-6.47) | 0.117 |
| Days from previous febrile neutropenia | 1.00 (1.00 -1.00) | 0.838 |
| Sum of days of neutropenia (previous 3 mo) | 1.02 (1.00-1.04) | 0.108 |
| Neutropenia at onset of BSI | 4.72 (0.61-36.9) | 0.139 |
| Neutrophils at the onset of bacteriemia <100/mm^3^ | 3.73 (0.82-16.9) | 0.089 |
| Days of neutropenia prior to onset of BSI | 1.00 (0.98-1.02) | 0.871 |
| Breakthrough febrile neutropenia^a^ | 11.93 (3.5-41.1) | < 0.001 |
| Prior antibiotic therapy (within 1 mo) | 3.38 (1.19-9.61) | 0.022 |
| Total number of days of systemic antibiotic therapy (1 mo) | 1.08 (1.03-1.15) | 0.005 |
| Days from last systemic antibiotic therapy (1 mo) | 0.92 (0.81-1.03) | 0.139 |
| Prior carbapenem therapy (1 mo) | 4.76 (1.65-13.7) | 0.004 |
| Total number of days of carbapenem therapy (1 mo) | 1.16 (1.03-1.30) | 0.013 |
| Colonization with multidrug-resistant GNB (6 mo) | 0.73 (0.09-6.04) | 0.768 |
| Previous isolations of MDR-GNB in samples other than colonization samples (6 mo) | 4.73 (1.31-17.11) | 0.018 |
| BSI = bloodstream infection; CS = carbapenem susceptible; CR = carbapenem resistant; GNB = Gram-negative bacteria; MDR, multi-drug resistant; HSCT, Hematopoietic stem cell transplantation.  ^a^ During the episode of febrile neutropenia, the patient presents with fever and clinical fever while still receiving systemic antibiotics at therapeutic doses or has received them in the previous 7 days. | | |

| **Table S7**. Univariate analysis of baseline variables associated with carbapenem‑resistant Gram‑negative bloodstream infections in the cohort of patients with febrile neutropenia | | |
| --- | --- | --- |
|  | **OR (95% CI)** | **p** |
| Sex (male) | 1.52 (0.42-5.55) | 0.529 |
| Age (years) | 0.98 (0.94 – 1.03) | 0.467 |
| Hematological disease |  |  |
| Acute leukemia/myelodysplastic syndrome | 1.60 (0.47-5.44) | 0.45 |
| Lymphoma | 0.25 (0.051-1.26) | 0.094 |
| Multiple myeloma/Waldenström disease | 3.14 (0.18-53.6) | 0.429 |
| Other | 3.31 (0.42-25.8) | 0.254 |
| Hematological disease status |  |  |
| First line of treatment | 1.09 (0.29-4.14) | 0.898 |
| Refractory disease | 2.30 (0.55-9.64) | 0.254 |
| 1^st^ complete remission | 0.40 (0.11-1.44) | 0.161 |
| ≥2^nd^ complete remission | 6.62 (0.55-78.9) | 0.135 |
| Others | 0.56 (0.06-5.19) | 0.608 |
| HSCT | 0.50 (0.03-7.54) | 0.617 |
| ECOG Performance Status Scale upon admission >=3 | 16.0 (1.62-157.8) | 0.018 |
| Charlson comorbidity index | 1.31 (0.83-2.07) | 0.243 |
| MASCC index < 21 | 2.29 (0.67-7.76) | 0.185 |
| Septic shock at FN episode onset | 1.79 (0.81-3.98) | - ^a^ |
| FN episode onset in an ambulatory setting | 0.42 (0.08-2.16) | 0.301 |
| Days of admission before FN episode onset | 1.04 (0.99-1.09) | 0.140 |
| Prior hospital admission (within 3 mo) | 0.38 (0.11-1.33) | 0.129 |
| Total number of days of prior hospital admission (3 mo) | 1.01 (0.98-10.3) | 0.586 |
| Previous neutropenia (previous 90 days) | 0.81 (0.14-4.70) | 0.817 |
| Previous FN | 1.31 (0.40-4.30) | 0.654 |
| Days from previous FN | 0.99 (0.98-1.02) | 0.931 |
| Sum of days of neutropenia (previous 3 mo) | 1.01 (0.988-1.04) | 0.321 |
| Neutrophils at the onset of FN episode <100/mm^3^ | 1.13 (0.48-2.68) | - ^a^ |
| Days of neutropenia prior to onset of FN episode | 1.00 (0.99-1.02) | 0.668 |
| Breakthrough FN ^b^ | 1.64 (0.49-5.55) | 0.425 |
| Prior antibiotic therapy (within 1 mo) | 1.20 (0.37-3.85) | 0.766 |
| Total number of days of systemic antibiotic therapy (1 mo) | 1.06 (0.99-1.13) | 0.105 |
| Prior carbapenem therapy (1 mo) | 1.00 (0.31-3.23) | 1.000 |
| Total number of days of carbapenem therapy (1 mo) | 0.99 (0.88-1.14) | 0.987 |
| Colonization with multidrug-resistant GNB (6 mo) | 0.39 (0.04-3.44) | 0.395 |
| Previous isolations of MDR-GNB in samples other than colonization samples (6 mo) | 2.00 (0.42-9.61) | 0.387 |
| Composite variable for the identification of CR-GNBSI | 7.43 (1.50-38.8) | 0.014 |
| BSI = bloodstream infection; CS = carbapenem susceptible; CR = carbapenem resistant; FN = febrile neutropenia; GNB = Gram-negative bacteria; MDR, multi-drug resistant; HSCT, Hematopoietic stem cell transplantation.  ^a^ Due to perfect collinearity in this predictor, Ridge regression was applied to stabilize the estimation of the coefficients. The OR and its 95% CI were then derived from the penalized model. This method does not yield a conventional p-value.  ^b^ During the episode of febrile neutropenia, the patient presents with fever and clinical fever while still receiving systemic antibiotics at therapeutic doses or has received them in the previous 7 days. | | |

**Figure S1**. ROC curves in quantitative variables predictors of CR-GNBSI in the cohort of hematological patients with GNBSI

CR = Carbapenem-resistance; GNBSI = Gram-negative bloodstream infection; ROC = receiver operating characteristic.
